# What opportunities do early career psychiatrists have in Europe and beyond?

**DOI:** 10.1192/bji.2020.29

**Published:** 2020-11

**Authors:** Mariana Pinto da Costa, Ozge Kilic, Jamila Ismayilova, Tove Mogren, Daria Smirnova, Tomasz M. Gondek

**Affiliations:** 1 Unit for Social and Community Psychiatry (WHO Collaborating Centre for Mental Health Services Development), Queen Mary University of London, UK; 2 Department of Psychiatry, Koc University Hospital, Istanbul, Turkey; 3 National Mental Health Center, Baku, Azerbaijan; 4 Department of Psychiatry, Psykiatricentrum Södertälje, Stockholm, Sweden; 5 Department of Psychiatry, Samara State Medical University, Russia; 6 Polish Psychiatric Association, Specialty Training Section, Wroclaw, Poland. Email: tomaszmgondek@gmail.com

**Keywords:** Education and training, professional development, organisations, early career, collaborative research

## Abstract

The European Psychiatric Association (EPA), the main association in the field of mental health in Europe, has long been supporting the development of early career psychiatrists. The EPA Early Career Psychiatrists Committee (ECPC) and its core task forces promote research activities among young psychiatrists, contribute to their professional development through organising courses and other educational events, prepare online educational materials and publications, and actively collaborate with other organisations. The EPA ECPC is always open to fostering cooperation on new professional, educational or research initiatives with early career psychiatrists from different countries.

## Introduction

Over the past century, the integration of a wide range of biological, psychological and social sciences has led to significant advances in psychiatry, making it a broad medical specialty closely linked to somatic medicine, psychology and society. Many psychiatrists early in their career have understood that it is easier to face challenges in a collaborative way; in recent decades, local and national associations of psychiatric trainees and early career psychiatrists have emerged and grown across Europe and beyond.

The European Psychiatric Association (EPA, formerly known as the Association of European Psychiatrists) was founded in 1983 in Strasburg, and the first European Congress of Psychiatry was organised in 1986. Today, with individual members from 88 countries and with 44 National Psychiatric Association members representing more than 80 000 psychiatrists, the EPA is the leading association in the field of mental health in Europe. The association supports psychiatrists at all career stages in their clinical practice, scientific research and teaching activities. Importantly, much attention is paid to the development of those early in their career.

## Who is an early career psychiatrist?

Within the EPA, it is possible to join the network of early career psychiatrists if you are a trainee in psychiatry, if your work experience does not exceed 5 years from completion of professional training, or if your age is under 40. Membership in the network helps promote fruitful cooperation with other young psychiatrists with similar education and research interests. The EPA assists early career psychiatrists in developing networks, mentoring and academic opportunities. Furthermore, the EPA provides young psychiatrists with an opportunity to voice their opinions about training, research and practice standards, thereby empowering them in their professional development and career progression.

## The beginnings of the Early Career Psychiatrists Committee

In 2007, the EPA integrated into its programme the Young Psychiatrists Committee, which is an informal network of young psychiatrists, many of them past representatives of the European Federation of Psychiatric Trainees (EFPT). Since 2010, this committee has been known as the Early Career Psychiatrists Committee (ECPC).

In 2011, the ECPC formulated a biennial action plan with the aim of promoting the participation of young psychiatrists in the European Congress of Psychiatry and various other activities dedicated to their needs. The purpose of this action plan was to identify and effectively tackle problems faced by psychiatrists during the early stages of their careers. It also aimed to design new activities to promote the professional development of young psychiatrists, and to propose a new agenda for the training of early career psychiatrists in Europe. During the year of its inception, the ECPC created the self-rated Psychiatric Training Questionnaire, which enquires about the following areas: satisfaction with training; self-confidence in various fields of clinical psychiatry; participation in research activities; and, finally, the compatibility of training experiences with the standards set by the Union of European Medical Specialists (UEMS) Section of Psychiatry.

The EPA has made substantial efforts to support quality education for trainees during their early career and to facilitate their participation in scientific congresses, thereby promoting their professional growth and integration into the international professional community. Likewise, early career psychiatrists have contributed to the EPA with their energy, motivation, enthusiasm and creative ideas, supporting this initiative with their effective cooperation.

## Current activities

The ECPC's activities are encompassed by four core task forces – research, communication and publications, professional development, and meetings and associations. The Research Task Force aims to promote international scientific projects in European countries and to help young psychiatrists to develop research skills. It provides assistance and signposting to the necessary resources to launch research activities, and has published numerous articles in peer-reviewed scientific journals. The Communication and Publications Task Force focuses on various online educational materials posted on the official EPA website. One major outcome has been the publication of two books written by early career psychiatrists. The Professional Development Task Force cooperates with various organisations to hold courses, workshops, seminars and other educational activities, including the Gaining Experience programme. In addition, this task force conducts surveys to assess the needs of early career psychiatrists.

Last but not least, the Task Force on Meetings and Associations collaborates actively with other organisations, including the Early Career Psychiatrists section of the World Psychiatric Association and the EFPT. It liaises with other early career psychiatrists, inviting them to participate in the EPA Congress and in other collaborative professional meetings and events. It also represents the ECPC at meetings, including those organised by the UEMS, and has promoted ECPC's activities in our partners’ newsletters.

## Opportunities

The ECPC's objectives are also achieved through the organisation of a high-quality scientific discussion group for early career psychiatrists within the annual EPA Congress, where young colleagues have the opportunity to share ideas and experiences and inspire others. We are proud to report that many internationally renowned senior psychiatrists greatly enjoy participating in these activities. Importantly, since 2007, a scholarship programme has been in place for European early career psychiatrists, supporting their registration, travel and accommodation expenses at the EPA Congress.

The ECPC is also proud to have initiated the Gaining Experience programme, a scholarship that funds brief observership placements (between 2 and 8 weeks) in various psychiatric institutions across Europe. The scholarship provides an opportunity for early career psychiatrists who have finished their psychiatric training to broaden their clinical, research or teaching skills and their knowledge.

For some years, the ECPC has also supported the Committee of Education in the preparation of the EPA Summer School. This is an annual educational meeting for young psychiatrists that has focused on a range of topics including comorbidities between mental and somatic disorders, the ‘ABCs’ of psychotherapy and, more recently, research.

## Associations and collaborations

The ECPC has collaborated on various projects with other international organisations. It is open to developing new professional, educational and research initiatives with other organisations or individual early career psychiatrists from different countries. Given the opportunities that having a local association dedicated to early career psychiatrists can provide, the ECPC Task Force on Meetings and Associations is also ready to help colleagues to establish new associations for early career psychiatrists in countries where there is no such organisation.

Local and national associations may play a key part in promoting quality improvement of psychiatric training and improving the working conditions of psychiatric trainees and young specialists. They may also facilitate the establishment or strengthening of peer networks through organising educational and scientific events that bring early career psychiatrists together.

The first step to starting an association is finding others who share an identity and similar values and are willing to join forces. Early career psychiatrists who set up such an association will become its founding members; they can form collaborations with other colleagues locally and can be influential in shaping organisational structures. The ECPC is always glad to be contacted and assist those who wish to set up a framework for a new association dedicated to early career psychiatrists. By organising ourselves, we can make our voice better heard, work collaboratively towards our goals and celebrate together our joint achievements.

## Contacting the ECPC

The EPA ECPC is open to being contacted by early career psychiatrists who are willing to contribute to developing activities. Please contact ecpc@europsy.net. Further information and subscription to the Early Career Psychiatrists mailing list is available at www.europsy.net/early-career-psychiatrists-committee.

## Supplementary material

For supplementary material accompanying this paper visit https://doi.org/10.1192/bji.2020.29. click here to view supplementary material

